# Affect-Modulated Startle: Interactive Influence of Catechol-*O*-Methyltransferase Val158Met Genotype and Childhood Trauma

**DOI:** 10.1371/journal.pone.0039709

**Published:** 2012-06-22

**Authors:** Benedikt Klauke, Bernward Winter, Agnes Gajewska, Peter Zwanzger, Andreas Reif, Martin J. Herrmann, Andrea Dlugos, Bodo Warrings, Christian Jacob, Andreas Mühlberger, Volker Arolt, Paul Pauli, Jürgen Deckert, Katharina Domschke

**Affiliations:** 1 Department of Psychiatry and Psychotherapy, University of Muenster, Muenster, Germany; 2 Christoph Dornier Clinic of Psychotherapy, Muenster, Germany; 3 Department of Psychiatry, Psychosomatics and Psychotherapy, University of Wuerzburg, Wuerzburg, Germany; 4 Department of Psychology, University of Wuerzburg, Wuerzburg, Germany; Rikagaku Kenkyūsho Brain Science Institute, Japan

## Abstract

The etiology of emotion-related disorders such as anxiety or affective disorders is considered to be complex with an interaction of biological and environmental factors. Particular evidence has accumulated for alterations in the dopaminergic and noradrenergic system – partly conferred by catechol-*O*-methyltransferase (*COMT*) gene variation – for the adenosinergic system as well as for early life trauma to constitute risk factors for those conditions. Applying a multi-level approach, in a sample of 95 healthy adults, we investigated effects of the functional *COMT* Val158Met polymorphism, caffeine as an adenosine A2A receptor antagonist (300 mg in a placebo-controlled intervention design) and childhood maltreatment (CTQ) as well as their interaction on the affect-modulated startle response as a neurobiologically founded defensive reflex potentially related to fear- and distress-related disorders. *COMT* val/val genotype significantly increased startle magnitude in response to unpleasant stimuli, while met/met homozygotes showed a blunted startle response to aversive pictures. Furthermore, significant gene-environment interaction of *COMT* Val158Met genotype with CTQ was discerned with more maltreatment being associated with higher startle potentiation in val/val subjects but not in met carriers. No main effect of or interaction effects with caffeine were observed. Results indicate a main as well as a GxE effect of the *COMT* Val158Met variant and childhood maltreatment on the affect-modulated startle reflex, supporting a complex pathogenetic model of the affect-modulated startle reflex as a basic neurobiological defensive reflex potentially related to anxiety and affective disorders.

## Introduction

The etiology of anxiety and affective disorders is considered to be complex with an interaction of biological factors and environmental influences: Family and twin studies propose a genetic contribution to the pathogenesis of these disorders with an estimated heritability of 30 to 60% [1]–[3]. The remaining part of the variance has been attributed to environmental factors [3], [4].

Particular evidence is accumulating for the catechol-*O*-methyltransferase (COMT) as a promising candidate gene in the pathogenesis of anxiety and affective disorders: COMT catalyzes the inactivation of monoaminergic neurotransmitters, particularly dopamine and norepinephrine, by an extraneural transfer of a methyl group to catechol compounds. Significantly elevated erythrocyte COMT activity has been reported in patients with anxiety states [5] and COMT inhibitors are effectively used in the treatment of anxiety symptoms in Parkinson's disease [6]. A single nucleotide polymorphism (472G/A) in the *COMT* gene, located on chromosome 22q11.2 [7], causes an amino acid change from valine to methionine at position 158 (Val158Met), with the val allele (472G) conferring an at least 40% higher COMT activity [8], [9]. This more active val allele has been reported to be associated with panic disorder [10]–[13], phobic anxiety [14], neuroticism [15], harm avoidance [16] and generalized anxiety [17]. However, there are also reports indicating no influence of *COMT* Val158Met on anxiety disorders or related phenotypes [18]–[23] or demonstrating association of the less active met allele with anxiety-related phenotypes [24]–[32]. Association studies of the *COMT* Val158Met polymorphism with respect to affective disorders, in particular depression, are similarly inconclusive [33]–[35].

Three aspects might have to be taken into consideration in order to reconcile these inconsistent molecular genetic findings and to elucidate the genetic underpinnings of anxiety/affective disorders in a more comprehensive way: 1) intermediate phenotypes, 2) interaction of several relevant neurotransmitter systems and 3) interaction of genetic and environmental factors.

1. Specification to unravel the influence of genetic factors on complex traits or diseases can be reached by investigation of so-called endophenotypes on an intermediate level between genetic factors and categorical disease phenotypes [36]. The acoustic startle response and particularly the affect-modulated acoustic startle response are neurobiologically founded behavioral measures of emotional reactivity reflecting a defensive motivational state [37]–[46]. Accordingly, there is evidence for exaggerated startle potentiation in response to negative emotional stimuli in anxiety disorders [39]–[41], [46]–[48] and fear- or anxiety/distress-related states [37], [49]. Twin studies provide evidence for a genetic influence on different components of the startle reflex (heritability: ∼30–70%; [50]–[54]), with several studies having investigated the possible role of *COMT* gene variation: Montag et al. [55] found greater startle responses for met homozygotes in the unpleasant condition of an acoustic affect-modulated startle paradigm, while Pauli et al. [56] using the same paradigm failed to discern any influence of *COMT* gene variation on startle modulation. Armbruster et al. [57] discerned a significant *COMT* Val158Met genotype effect on average startle magnitudes across conditions with met/met carriers showing the highest and val/val homozygotes showing the lowest startle response, while no influence of *COMT* genotype on the emotional modulation of the startle reflex was detected. Lonsdorf et al. [31] and Klumpers et al. [58] did not discern any effect of *COMT* Val158Met on fear-potentiated startle during acquisition of fear conditioning or during instructed fear, respectively.
2. The dopamine/norepinephrine system, as crucially driven by the *COMT* Val158Met polymorphism, ought not to be considered in an isolated way with respect to the modulation of anxiety or related phenotypes, but rather in interaction with other relevant neurotransmitter systems. Animal and human studies have e.g. suggested a tight functional link between the dopamine and the adenosine system on a cellular as well as a neurotransmitter level [59]–[61]. Caffeine, which is an antagonist at the adenosine A2A receptor and acts as a potent anxiogenic and arousal-increasing substance [62], [63], has been reported to increase acoustic startle reflex amplitude and confer a delayed habituation of acoustic startle blink amplitude [64]–[66]. In addition, we have previously observed a significant interactive effect of adenosine A2A receptor gene variation, caffeine intervention and emotional stimuli on startle magnitudes [67]. The *COMT* Val158Met polymorphism as a potent genetic modulator of dopaminergic signaling on the one hand and caffeine administration on the other hand might thus serve as valid biological measures to investigate the interactive influence of the dopaminergic and the adenosinergic system on the affect-modulated startle response.
3. Given the complex-genetic nature of anxiety disorders entailing an interactive pathogenetic effect of genetic and environmental factors, neglect of environmental factors might have introduced a major bias to previous genetic studies. There is converging evidence for a crucial role of abuse [68]–[70] and loss/separation experiences [71], [72] on the pathogenesis of anxiety disorders [73], with childhood and adolescence being considered as particularly sensitive periods [74]. In mice, prolonged pre-pubertal stress enhanced the acoustic startle reflex [75]. Consistently, early environmental stressors such as perceived childhood physical and sexual abuse experiences have been shown to increase baseline startle reactivity in humans [76; but 77]. With respect to the interactive influence of *COMT* gene variation and life events in anxiety, only few gene-environment interaction (GxE) studies are available: Kolassa et al. [78] reported a GxE interaction of the Val158Met polymorphism and traumatic events in the etiology of posttraumatic stress disorder, with val allele carriers showing a trauma quantity dependent disease risk, while met homozygotes exhibited a high risk for PTSD independently of the severity of traumatic load. In contrast, no interaction effect was found between Val158Met and early adversity or stressor experiences on anxiety in young children [23] or anxiety-related traits [19], respectively.

Given these multi-level factors possibly modulating the influence of *COMT* gene variation on anxiety and affective disorders or related phenotypes, in the present study applying an integrative approach using the same study design as in Domschke et al. [67] we set out to elucidate the main as well as interactional effects of *COMT* gene variation, caffeine and childhood maltreatment on the affect-modulated startle response as a neurobiological measure of emotional and motivational processes potentially related to fear- and anxiety/distress-related states.

## Results

### Descriptive Data

Five of 95 examined participants showed too many zero startle responses (<5 µV; 2.5 standard deviations above mean value) and were therefore excluded from further analyses. For all affect-modulated startle responses, no outliers (>2.5 SD) were detected. For the mean intertrial interval (ITI) startle response, one subject was additionally excluded because of an extreme score (>2.5 SD), so that this analysis step was run with 89 participants only.

For age and CTQ, no differences were observed between sex (male vs. female probands), challenge conditions (caffeine vs. placebo) or *COMT* Val158Met genotype groups (data not shown). Descriptive statistics of age, CTQ and startle (ITI startle, startle after unpleasant and pleasant picture (International Affective Picture System; IAPS) presentation, startle magnitude potentiation after unpleasant picture presentation compared to neutral pictures [Diff_unpl-neutr_]) for the 90 probands are given in table 1.

**Table 1:** Descriptive characteristics of probands by sex, challenge condition and *COMT* Val158Met genotype.

				Male			Female			Caffeine			Placebo		
Variable	N	Mean	SD	N	Mean	SD	N	Mean	SD	N	Mean	SD	N	Mean	SD
Total sample															
Age (years)	90	26.46	6.22	45	27.00	5.93	45	25.91	6.51	43	25.98	5.29	42	26.60	6.80
CTQ (sum)	90	33.23	5.60	45	32.71	4.66	45	33.76	6.41	43	32.88	5.51	42	33.57	5.92
ITI startle	89	37.29	26.46	44	35.55	26.26	45	39.00	26.84	43	35.54	24.76	41	39.66	28.51
Startle unpl	90	51.38	2.62	45	50.57	2.26	45	52.19	2.73	43	51.45	3.03	42	51.24	2.19
Startle pl	90	48.64	1.79	45	48.91	1.50	45	48.38	2.02	43	48.68	1.89	42	48.81	1.69
Diff_unpl-neutr_	90	1.42	4.48	45	−0.01	3.90	45	2.85	4.61	43	1.58	5.16	42	1.30	3.76
Sample stratified by genotype															
***COMT*** ** val/val**															
Age (years)	15	25.47	4.87	6	26.17	4.54	9	25.00	5.29	7	26.29	4.35	6	23.17	0.98
CTQ (sum)	15	32.87	4.24	6	33.83	3.97	9	32.22	4.52	7	32.86	2.85	6	33.00	5.97
ITI startle	15	36.13	23.95	6	23.69	21.76	9	44.42	22.68	7	34.49	24.27	6	31.09	22.75
Startle unpl	15	53.16	2.14	6	52.72	0.99	9	53.45	2.68	7	53.50	2.48	6	52.77	2.23
Startle pl	15	48.02	1.69	6	48.23	1.07	9	47.87	2.05	7	48.17	1.29	6	48.46	2.07
Diff_unpl-neutr_	15	4.41	3.99	6	3.75	1.96	9	4.84	4.99	7	5.22	5.24	6	3.99	2.95
***COMT*** ** val/met**															
Age (years)	42	25.83	6.07	22	26.45	5.79	20	25.15	6.45	22	24.45	3.46	20	27.35	7.86
CTQ (sum)	42	33.40	6.01	22	33.27	5.13	20	33.55	6.98	22	33.05	6.27	20	33.80	5.85
ITI startle	41	34.00	19.95	21	34.60	18.38	20	33.36	21.95	22	30.61	15.89	19	37.91	23.66
Startle unpl	42	50.89	2.79	22	50.29	2.54	20	51.56	2.97	22	50.86	3.32	20	50.93	2.15
Startle pl	42	49.28	1.81	22	49.13	1.57	20	49.44	2.07	22	49.41	2.04	20	49.12	1.55
Diff_unpl-neutr_	42	1.00	4.73	22	−0.42	4.43	20	2.56	4.65	22	1.07	5.47	20	0.92	3.89
***COMT*** ** met/met**															
Age (years)	33	27.70	6.88	17	28.00	6.67	16	27.38	7.29	14	28.21	7.28	16	26.94	6.50
CTQ (sum)	33	33.18	5.75	17	31.58	4.26	16	34.88	6.72	14	32.64	5.54	16	33.50	6.37
ITI startle	33	41.92	33.77	17	40.90	34.62	16	43.01	33.95	14	43.80	34.44	16	44.95	35.50
Startle unpl	33	51.19	2.31	17	50.17	1.82	16	52.28	2.32	14	51.36	2.46	16	51.06	2.13
Startle pl	33	48.12	1.57	17	48.87	1.53	16	47.33	1.22	14	47.77	1.43	16	48.54	1.75
Diff_unpl-neutr_	33	0.59	3.90	17	−0.82	2.94	16	2.08	4.31	14	0.55	4.06	16	0.77	3.63

ITI startle  =  startle response during the intertrial interval in µV (ITI); Startle unpl  =  startle response after presentation of unpleasant IAPS pictures (in µV; T-transformed); Startle pl  =  startle response after presentation of pleasant IAPS pictures (in µV; T-transformed); Diff_unpl-neutr_  =  potentiation of startle magnitude by unpleasant IAPS pictures (in µV; T-transformed values: contrast unpleasant/neutral pictures); CTQ  =  Childhood Trauma Questionnaire (sum score).

Note: Because of the large variance of caffeine saliva concentrations (difference of more than one and a half interquartile ranges from the respective median in the placebo or verum group) five subjects were excluded, leaving 85 subjects for further analyses when considering challenge condition. For the mean ITI startle response, one subject was excluded because of an extreme score (> three SD).

In the present sample, CTQ sum scores ranged between a minimum of 26 and a maximum of 52 (mean = 33.23, SD = 5.60).

### Gene-Environment Correlation (rGE)

No significant gene-environment correlation between *COMT* Val158Met genotypes and CTQ was observed (rGE = −0.01, *p* = 0.92). Thus, confounding effects of a correlation between genetic and environmental predictors could be excluded.

### Influence of *COMT* Val158Met on baseline startle reflex

No influence of *COMT* Val158Met genotype on mean ITI startle response was observed (*F*(2,87) = 0.24, *p* = 0.79).

### Influence of *COMT* Val158Met, sex and challenge condition on affect-modulated startle

Because of the large variance of caffeine saliva concentrations especially in the placebo group (range: 0–132 mg/l), all subjects with a concentration differing more than one and a half interquartile ranges from the respective median in the placebo or verum group were excluded for analyses including challenge condition as an additional between-subjects factor. For this reason, five subjects in the placebo group had to be excluded, leaving 85 subjects for analyses including challenge condition (see table 1).

According to Mauchlýs test, sphericity assumption was violated (*χ^2^*(2) = 19.23, *p*<0.001), so that Huynh-Feld correction was used (ε = 0.95). In the presently analyzed sample of N = 85 probands, ANOVA revealed a significant effect of picture valence on startle magnitude (*F*(2,146) = 26.97, *p*<0.001), representing a linear trend (*F*(1,73) = 47.06, *p*<0.001) with increasing startle magnitudes from pleasant to unpleasant pictures.

Furthermore, significant interactions between *COMT* Val158Met genotype and picture valence (*F*(4,146) = 3.99, *p* = 0.005) and between sex and picture valences (*F*(2,146) = 4.43, *p* = 0.015) were observed. No significant interaction between challenge condition and picture valence (*F*(2,146) = 0.17, *p* = 0.83) and no significant 3- or 4-way-interactions between picture category and the between-subjects factors (*COMT* Val158Met genotype, sex, challenge condition) were observed, respectively (data not shown).

**Figure 1:**
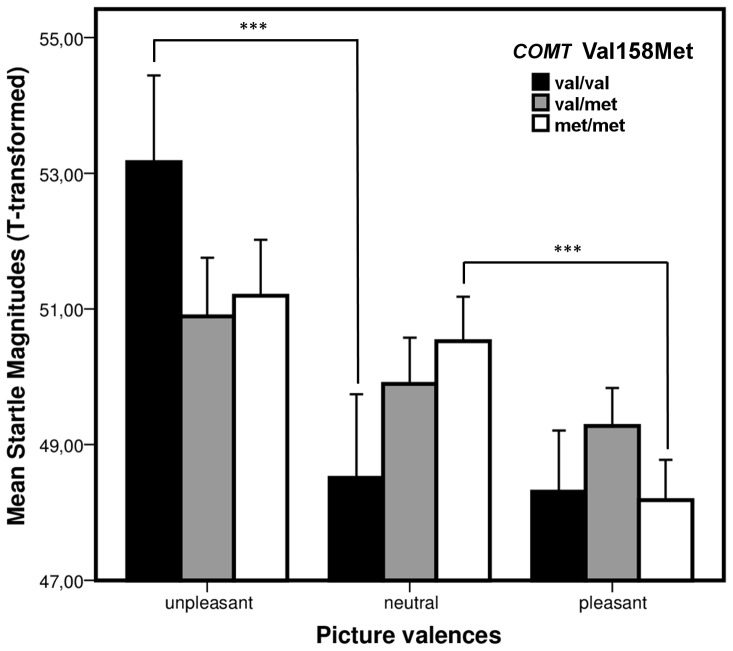
Mean startle magnitude modulated by picture category and *COMT* Val158Met genotype. *** significant at significance level of *p*≤.005.

Post-hoc t-tests revealed a significantly increased startle response to unpleasant pictures in comparison to neutral pictures in val homozygotes (*t*(12) = 3.98, *p* = 0.002), but not in met homozygotes (*t*(29) = 0.98, *p* = 0.34). Vice versa, a significantly decreased startle response to pleasant pictures compared to neutral pictures was observed in met homozygotes (*t*(29) = 5.28, *p*<0.001), but not in homozygotes for the val allele (*t*(12) = 0.24, *p* = 0.82). For carriers of the val/met genotype, no differences, neither in response to unpleasant compared to neutral pictures (*t*(41) = 1.37, *p* = 0.18) nor in response to neutral compared to pleasant pictures (*t*(41) = 1.47, *p* = 0.16) were observed (see figure 1).

Post-hoc t-tests stratified for sex revealed a significantly increased startle response in response to unpleasant pictures in comparison to neutral pictures in female subjects (*t*(39) = 4.22, *p*<0.001), but not in male participants (*t*(44) = −0.02, *p* = 0.98). A significantly increased startle response to neutral pictures compared to pleasant pictures was observed in male subjects (*t*(44) = 4.68, *p*<0.001), but not in female participants (*t*(39) = 1.18, *p* = 0.25).

Using untransformed raw startle data, the results remained stable, showing a significant effect of picture valence on startle magnitude, a significant interaction between *COMT* Val158Met genotype and picture valence and, a significant interaction between sex and picture valence (data not shown).

### Influence of *COMT* Val158Met and CTQ on baseline startle reflex

Investigating influences of CTQ on mean ITI startle response for each *COMT* Val158Met genotype separately, no associations were observed, neither for subjects carrying the *COMT* val/val genotype (*β* = −0.40, *t* = −1.59, *p* = 0.14), nor for val/met (*β* = −0.05, *t* = −0.29, *p* = 0.77) or met/met genotype carriers (*β* = −0.15, *t* = −0.83, *p* = 0.41).

### Influence of *COMT* Val158Met and CTQ on affect-modulated startle reflex

After presentation of unpleasant pictures, only for val homozygotes a significant influence of CTQ on startle response was observed: val/val carriers showed an increased startle when at the same time scoring high on the CTQ (*β* = 0.52, *t* = 2.18, *p*<0.05). No influence of CTQ on the startle reflex after unpleasant picture presentation was observed in val/met (*β* = −0.24, *t* = −1.55, *p* = 0.13) carriers or met homozygotes (*β* = 0.05, *t* = 0.30, *p* = 0.77).

After neutral picture presentation, startle responses for none of the *COMT* Val158Met genotype groups were influenced by CTQ (val/val: *β* = −0.15, *t* = −0.56, *p* = 0.59; val/met: *β* = 0.24, *t* = 1.54, *p* = 0.13; met/met: *β* = 0.12, *t* = 0.69, *p* = 0.50).

After pleasant picture presentation, a marginally significant decrease of the startle response was observed for val homozygotes dependent on an increase in CTQ scores (*β* = −0.51, *t* = −2.16, *p* = 0.05), while no associations were observed for val/met (*β* = 0.15, *t* = 0.97, *p* = 0.34) or met/met genotype carriers (*β* = −0.21, *t* = −1.17, *p* = 0.25), respectively.

**Figure 2:**
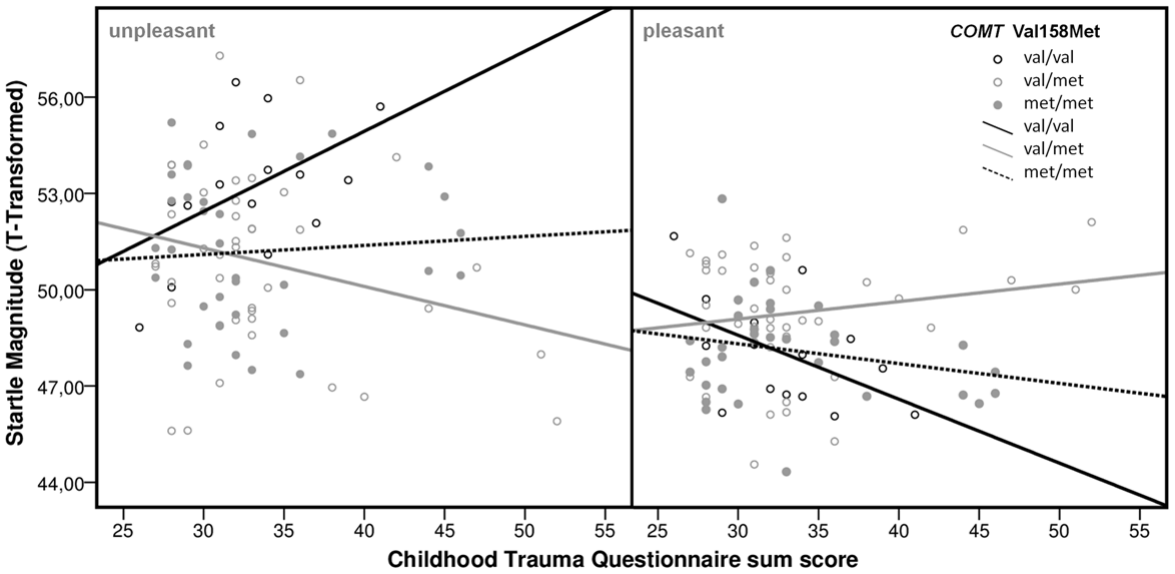
Linear regression analysis of CTQ sum score influence on startle magnitude potentiation after unpleasant and pleasant IAPS pictures stratified by *COMT* Val158Met genotype.

The interaction of *COMT* Val158Met genotypes and CTQ scores after presentation of unpleasant and pleasant pictures, respectively, is shown in figure 2.

### Hierarchical Multiple Regression

Based on the above mentioned results, influences of *COMT* Val158Met genotype, sex and CTQ as well as their interactive influence on startle magnitude potentiation after unpleasant IAPS picture presentation compared to neutral pictures (Diff_unpl-neutr_) were tested. Additionally, based on the observed results (see figure 2), *COMT* Val158Met genotypes were grouped reflecting a recessive model for the val allele (*COMT* val/val vs. met allele carriers).

In step 1, significant main effects of sex and *COMT* Val158Met genotype were observed, while CTQ alone did not affect startle magnitude potentiation. In step 2, in addition to the main effects of sex and *COMT* Val158Met genotypes, a significant interaction between *COMT* Val158Met genotypes and CTQ on startle magnitude potentiation (Diff_unpl-neutr_) was discerned. The addition of a GxE interaction term in step 2 accounted for a significant increment in explained variance. The inclusion of interactions with sex in step 3, did not significantly increase the explained variance. No significant interaction terms with sex were observed (see table 2).

**Table 2:** Results of hierarchical multiple regression analysis regarding affect-modulated startle magnitude (contrast unpleasant/neutral IAPS pictures; Diff_unpl-neutr_).

				Step 1 Statistics		Step 2 Statistics		Step 3 Statistics	
Predictors	*R* ^2^	Δ*R* ^2^	Δ*F* (*p*)	*β*	*t*	*β*	*t*	*β*	*t*
**Step 1**	.191	**.191** **	**6.75 (.001)**						
sex				.31	**3.13** **	.33	**3.42** **	−6.10	−1.62
* COMT* (val/val)				.27	**2.78** **	−5.28	−2.04*	−3.83	−1.36
CTQ				−.12	−1.20	.14	.90	.02	.13
**Step 2**	.232	.041*	**4.59 (.035)**						
* COMT* (val/val) x CTQ						5.57	2.14*	4.15	1.48
**Step 3**	.263	.030	1.13 (.342)						
* COMT* (val/val) x sex								−4.23	−1.12
CTQ x sex								6.37	1.69
* COMT* (val/val) x CTQ x sex								4.14	1.10

*COMT* (val/val)  =  val/val vs. val/met and met/met genotypes; CTQ  =  Childhood Trauma Questionnaire (logarithmized sum score); *R* ^2^  =  coefficient of determination (explained variance); Δ*R* ^2^  =  increase of explained variance; Δ*F (p)*  =  change and significance of F-statistics; *β*  =  standardized regression coefficient; *t*  =  t-statistics.

** p<0.01 (bold); * p<0.05 (bold).

Post-hoc t-tests of the main effects revealed a significantly increased startle magnitude potentiation in female subjects compared to males (*t*(88) = 3.18, *p* = 0.002) and in val homozygotes compared to met allele carriers (t(88) = 2.95, p = 0.004).

Neither main nor interactive effects of *COMT* Val158Met genotype, sex and CTQ were observed on startle magnitude differences after neutral IAPS picture presentation compared to pleasant pictures (Diff_neutr-pl_) (data not shown). Accordingly, neither the GxE interaction term in step 2 (Δ*R* ^2^ = 0.000, Δ*F*(1,85) = 0.03, *p* = 0.87) nor interactions with sex in step 3 (Δ*R* ^2^ = 0.014, Δ*F*(3,82) = 0.40, *p* = 0.76) significantly increased the explained variance.

## Discussion

In the present study, we observed a significant influence of *COMT* Val158Met genotype as well as an interactive effect of *COMT* Val158Met genotype and childhood maltreatment (CTQ) on the affect-modulated startle response: 1) Subjects homozygous for the more active val allele showed significant startle potentiation in response to unpleasant pictures, while met allele homozygotes displayed a blunted startle response to unpleasant stimuli and a significantly decreased startle response to pleasant stimuli. 2) Subjects homozygous for the val allele and at the same time scoring high on the CTQ showed increased potentiation of the startle magnitude after unpleasant picture presentation and a significantly decreased startle response to pleasant stimuli. 3) No influence of caffeine on affect-modulated startle responses dependent on the *COMT* Val158Met polymorphism could be discerned.

Ad 1) We discerned significant startle potentiation in response to unpleasant stimuli in homozygous carriers of the more active *COMT* val allele. This pattern corresponds to the expected modulation of the startle response by aversive stimuli constituting an inherently defensive response [38], [44] reported in both healthy probands [44], [45] as well as in anxiety and anxiety disorders [39]–[41], [46]–[48]. Val allele carriers did not show significant modulation in response to pleasant pictures, potentially due to a relatively decreased response to neutral stimuli (see figure 1). This lack of startle modulation by positive pictures, however, is not uncommon and not as reliably replicated as the startle potentation in response to aversive stimuli [37].

Some support for a modulatory influence of the val allele on startle potentiation by aversive stimuli as presently observed might be derived from studies reporting *COMT* val/val genotype to decrease prepulse inhibition (PPI) of the startle reflex [79], [80]. Also, using the COMT inhibitor tolcapone, Giakoumaki et al. [81] observed lowered prepulse inhibition in the placebo condition and increased prepulse inhibition in the tolcapone condition in val homozygotes. Diminished PPI has been observed in aversive contexts when using pictures as both prestimuli and affective prompts [82] and along with increased fear-potentiated startle in patients with anxiety disorders [43], [83]–[85]. However, there is experimental evidence for valence modulation and prepulse inhibition to constitute completely independent startle modulatory phenomena [86], thus rendering hypotheses regarding the relation between the genetic modulation of affect-modulated startle on the one hand and prepulse inhibition one the other hand highly speculative.

The neurobiological circuit underlying the defensive response of increased startle response to aversive stimuli has been postulated to critically involve input from the amygdala [38], [87]. The *COMT* val allele has previously been shown to be associated with increased amygdala activation during processing of aversive stimuli such as fearful faces in healthy probands as well as in patients with panic disorder [88]–[91]. Thus, increased excitability of the amygdala in val allele carriers might constitute one of the neurobiological underpinnings of the presently observed startle potentiation in response to aversive stimuli. It has to be noted, though, that the impact of *COMT* gene variation on amygdala response to aversive stimuli is highly controversial with several contradictory reports of association [92]–[94]. Research findings on the role of the dopaminergic and noradrenergic system, crucially driven by COMT activity, in mediating emotional processing is also not unequivocal: reports of increased norepinephrine availability to reverse the negative bias in information processing characterizing mood and anxiety disorders and to reduce amygdala response to fearful faces [95], [96] support the present finding of the more active *COMT* val allele – conferring decreased norepinephrine availability – to increase the defensive startle reaction to aversive emotional stimuli. Conversely, with respect to the dopaminergic system most evidence rather points to an increase in dopaminergic signalling to enhance limbic response to unpleasant stimuli [97], [98].

Homozygosity for the less active *COMT* met allele, conferring increased dopamine and norepinephrine availability, was presently associated with a blunted affective startle response to unpleasant stimuli and a significantly decreased startle response to pleasant stimuli in contrast to neutral pictures. This pattern corresponds to a study by Forbes et al. [99] showing greater startle during the neutral condition than during the pleasant condition, but no increase in startle during the unpleasant condition in patients with unipolar depression. Also, several other studies provide evidence for a diminished fear potentiated startle in depression-related states [100]–[102] and in patients with anxiety disorders and comorbid depression [103]–[105].

It has been suggested that affect-modulated startle response patterns might aid in a more differentiated understanding of the neurobiological underpinnings of anxiety disorders and depression, with enhanced fear-potentiated startle prevailing in patients with anxiety disorders and diminished affect modulation of the startle in patients with depression [49]. Thus, *COMT* gene variation might constitute one of the neurobiological mechanisms of this differentiation with the more active val allele potentially increasing defense reflexes and thereby conferring a higher risk for anxiety-related traits or disorders, respectively, while the less active met allele seems to be rather associated with defensive responding deficits and thereby traits or disorders on a depressive continuum. It has to be noted, though, that categorical association studies of *COMT* gene variation in both anxiety disorders [10]–[15], [24], [25], [28]–[30], [106] and depression [33]–[35] yielded inconclusive results with respect to the direction of allelic association. Also, in the present study dimensional measures of anxiety or depressive traits were not taken into consideration, which therefore does not allow for inferences on the relation of the present results to psychopathology. However, future studies investigating genetic effects on the affect-modulated startle response as an intermediate phenotype reflecting emotional reactivity in combination with assessment of psychometric correlates of emotion-related mental disorders might aid in a more neurobiologically informed understanding of the fear-anxiety-distress spectrum.

The present results are in contrast to a study by Montag et al. [55], who reported increased startle reflexes for met homozygotes in the unpleasant condition of an acoustic affect-modulated startle paradigm, and Pauli et al. [56] and Armbruster et al. [57], who failed to discern any influence of *COMT* gene variation on the emotional modulation of the startle reflex in healthy probands. These studies differ from the present one in several aspects: Montag et al. [55] investigated a sample of female probands only, who were controlled for their hormonal status, while in the present study both sexes were included and menstrual cycle phase was not ascertained. This might have accounted for differing startle responses across studies [107], particularly, as an estrogenic response element in the *COMT* gene promoter region might render COMT expression particularly dependent on estrogen levels [108]. Also, in the study by Montag et al. [55] probands were pre-stratified not only for the *COMT* Val158Met polymorphism, but also for the *DRD2/ANKK1* Taq IA SNP, which could have fundamentally influenced their results in an epistatic way. The two studies failing to discern an effect of *COMT* gene variation on affect-modulated startle response investigated probands at an older age (m: 35.16±10.29 years, f: 35.00±10.18 years, [56]; 61.13±2.57 years, [57]) than the study by Montag et al. [55] (22.11±3.29 years) and the present one (26.42±6.11 years), which might have influenced the respective results, as an age-related decrease in emotional recognition and processing has been observed ([109], but: [110]).

Ad 2) Besides several possible explanations for diverging association results of *COMT* Val158Met with startle response and anxiety-/depression-related phenotypes as detailed above, the present study might contribute to further delineating the functional effect of *COMT* gene variation on vulnerability towards fear/anxiety/distress-related states by complementing molecular genetic information with environmental data. We identified significant gene-environment interaction of *COMT* Val158Met genotype with CTQ, with more maltreatment being associated with higher startle potentiation in val/val subjects but not in met carriers. This finding could reflect a possibly val allele driven inclination of traumatized individuals to experience excessive negative emotions, which would be in line with Kolassa et al. [78] reporting val allele carriers to display a trauma quantity dependent disease risk for posttraumatic stress disorder. Additionally, we observed more childhood trauma to predispose homozygous val allele carriers to an accentuated startle inhibition in response to pleasant pictures. This might point to a capacity of val allele carriers to nevertheless experience positive emotions as calming and safety-indicating. It has been suggested that after traumatic experiences positive emotions could buffer against depression by “correcting”, “restoring” or “undoing” the effects of negative emotions (e.g., [111], [112]). Based on this hypothesis, the *COMT* val allele in interaction with traumatic experiences could predispose to an increased risk of exaggerated negative emotional processing potentially predisposing to anxiety disorders such as PTSD (cf. [78]), while at the same time conferring resilience against depression by restoring autonomic quiescence following positive emotional stimuli (see [113]). However, as in the present study only healthy probands were investigated and no measures of coping strategies or other relevant psychometric measures were ascertained, future studies will have to probe his hypothesis.

The present results are in contrast to a previous study by Jovanovic et al. [76], who failed to discern an influence of early environmental stressors on the degree of fear-potentiated startle. As this study, however, did not include genetic information potentially mediating the impact on early life stress on the affect-modulated startle response, studies are not fully comparable. Gene-environment interaction studies on the effect of *COMT* gene variation on stress-related disorders are quite controversial: No interactional impact of *COMT* Val158Met and life events could be discerned on anxiety and depression in 7–8 years old children [23] or on extraversion and neuroticism among adults, respectively [114]. The *COMT* met/met genotype has, however, been shown to interact with maternity stressors in postpartum depression [115] and with stressors within the year preceding the onset of the first mood disorder episode in depressive adults [116]. Furthermore, carriers of both the *5-HTTLPR* S and *COMT* met alleles exhibited the greatest depressive response to chronic stress in a three-way G x G x E interaction [117]. A possible explanation for this flip-flop phenomenon, i.e., opposite direction of allelic association across studies (cf. [118]), might be that different life events at different ages might differentially interact with dopaminergic tone in shaping the risk for different psychopathological states: As it has been shown that early life stress comparable to the presently investigated childhood traumata leads to decreases in the levels of norepinephrine and dopamine in the frontal cortex and metabolites of dopamine and serotonin in the amygdala (cf. [119]), carriers of the more active *COMT* val allele entailing a lowered noradrenaline and dopamine tonus might be particularly susceptible to the development of mental disorders related to childhood trauma. Additionally, linkage disequilibrium with other relevant polymorphisms, gene-gene interactions or epigenetic modifications following early environmental stress (see [120]) have to be taken into consideration when interpreting the present finding. Still, the present pilot data might foster future gene-environment interaction studies in fear-, anxiety- or distress-related disorders contributing to further disentangling their complex genetic nature (e.g., [121]–[123]).

Ad 3) No statistically significant influence of challenge condition (placebo/caffeine) on affect-modulated startle responses dependent on the *COMT* Val158Met polymorphism and/or childhood trauma could be discerned. Also, genotype distribution of the adenosine 2A receptor (*ADORA2A*) 1976T>C (rs5751876, formerly 1083T>C, Tyr/Tyr) variant, a silent polymorphism in exon 2 of the *ADORA2A* gene, which has previously found to be associated with panic disorder and anxiety-related traits [124]–[128], did not differ across *COMT* Val158Met genotype groups. The present results thus do not support a COMT driven interaction between the adenosinergic and the dopaminergic system in the mediation of stress and affect-modulated startle response. However, this interaction might not primarily be conferred by *COMT*, but rather by dopamine D2 receptor (*DRD2*) gene variation, as DRD2 receptors in the amygdala have been suggested to play a role in setting up adaptive responses to cope with aversive environmental stimuli [129] and to functionally interact with adenosine receptors on a cellular level [130].

In addition to the caveats mentioned above, the following limitations of our study have to be considered when interpreting the present results: First of all, the present sample size particularly regarding the val/val genotype group was limited. Despite there was sufficient power (>0.90) to explain 23% of startle magnitude variance with four predictors in step 2 with a type I error rate of 0.05 according to a post-hoc power calculation for multiple regression using G*Power calculation software available online (University of Duesseldorf, Germany; http://www.psycho.uni-duesseldorf.de/abteilungen/aap/gpower3/), we cannot exclude false positive or false negative results, respectively. Particularly, with respect to challenge condition or sex subgroups, sample sizes are very small and might not allow for sufficient power, although a separate analysis conducted in the placebo group only revealed the same effects as in the total sample and therefore might be taken as some confirmation of our overall results. However, the present results have to be considered pilot data, which warrant replication in larger independent samples. Furthermore, with a mean age of 26.5 years our final sample is relatively young, so that a potentially confounding neglect of genetic predisposition for mental disorders manifesting at a later age cannot be excluded. Also, the average sum score of the CTQ in the presently investigated sample is low, indicating a small quantity of childhood maltreatment experiences and thus potentially super-normality of the present sample. Childhood maltreatment was assessed retrospectively, entailing impaired accuracy and reliability of answers due to recall bias or false answers (cf. [131]). Moreover, the CTQ is designed as a multi-factorial questionnaire [132], [133], so that subdimensions of maltreatment (e.g., emotional abuse, sexual abuse, etc.) might even more specifically interact with genetic factors in the etiology of anxiety. Future studies will therefore require more complex models of mediating and moderating factors between genes and the investigated phenotype, particularly given that personality and behavioral characteristics such as neuroticism or cognitive appraisal might moderate the GxE influence on the pathogenesis of anxiety and related intermediate phenotypes (cf. [73]).

In summary, the present results indicate a main as well as a GxE effect of the *COMT* Val158Met variant and childhood maltreatment on the affect-modulated startle reflex, supporting a complex pathogenetic model of the affect-modulated startle reflex as a basic neurobiological defensive reflex potentially related to anxiety and affective disorders. Future, preferably longitudinal studies investigating genetic and environmental effects on the affect-modulated startle response reflecting emotional reactivity might aid in a more neurobiologically informed understanding of the fear-anxiety-distress spectrum.

## Methods

### Samples and Procedures

A sample of unrelated healthy participants (N = 95; male = 46, female = 49; mean age: 26.42 years, SD = 6.11) was consecutively recruited at the Departments of Psychiatry, Universities of Muenster and Wuerzburg, Germany, in the context of a collaborative research study. Inclusion criteria were European descent (self-report up to 3^rd^ generation), right-handedness and fluency in German. Exclusion criteria were manifest mental axis I disorder (M.I.N.I.: [134]), pregnancy or breast feeding, severe medical conditions, use of illegal drugs (assessed by a urine drug screening), alcohol consumption of more than 140 g per week, daily smoking of more than 20 cigarettes a day, caffeine or lactose intolerance, high caffeine consumption (more than 3 cups of coffee per day), less than a high school education, and age under 18 and over 50 years. A blood sample (20 ml EDTA blood) was taken for genetic analysis. Participants were asked to refrain from caffeine or tea consumption for one week prior to the investigation and not to smoke, consume alcohol (assessed by a breath test) or take any medication for at least 24 h prior to the investigation. To exclude any neurological or other somatic disorders participants underwent a brief physical and neurological examination in a screening session one week before the startle-experiment, where additionally heart activity (electrocardiogram) and basic blood parameters were checked. The protocol was approved by the ethics committees of the Universities of Muenster and Wuerzburg, Germany, and written informed consent was obtained from all subjects during the screening session. The study has been conducted according to the principles expressed in the Declaration of Helsinki.

### Genotyping

All participants were genotyped for the *COMT* Val158Met polymorphism according to published protocols [10], [88]. Genotypes were determined by investigators blinded for outcome measures (startle reflex). Hardy-Weinberg criteria, assessed with the online available program DeFinetti (Wienker and Strom, accessed February 2011), were fulfilled for *COMT* Val158Met genotype distribution in the present sample (val/val: 17%, val/met: 47%, met/met: 36%; *p* = 0.792). The present sample constitutes a subsample (due to missing data for the environmental variable or *COMT* genotype) of participants included in a larger study [67], which was originally stratified for adenosine A2A receptor (*ADORA2A*) gene variation. As we have previously observed a significant interactive effect of *ADORA2A* 1976T>C genotype, caffeine intervention and picture category in the extended sample [67], *ADORA2A* 1976T>C genotypes were retrieved for also for the present sample (see [67]). Furthermore, as there is accumulating evidence for variation in the serotonin transporter gene (*5-HTTLPR*) to influence fear-potentiated startle response [92] and to interact with stressful life events to influence startle response or anxiety sensitivity [122], [135], we additionally genotyped our sample for this variant according to published protocols with minor modifications (see [136], [137]). Hardy-Weinberg criteria were fulfilled for both *ADORA2A* 1976T>C and *5-HTTLPR* genotype distribution (both p>0.05).

### Affect-modulated startle paradigm

After screening negative for drugs and pregnancy, all electrodes were fixed and checked for impedances below 5 kΩ. At first, eight startle stimuli (random intervals of one second till twelve seconds; 50 ms of 95 dB white noise with an instantaneous rise-time presented via Bose® Around-Ear Headphones) were presented, to get participants accustomed to the startle procedure and to minimize outlier startle responses during the critical trials. The experiment consisted of three blocks à 24 pictures and three minute breaks between the blocks. Each block contained eight unpleasant, eight neutral, and eight pleasant environmental cues, derived from a total of 72 pictures from the International Affective Picture System (IAPS: [138]); 24 of each valence, respectively). Pictures were randomized with the constraint that not two of the same valence (unpleasant, neutral or pleasant) were presented successively. IAPS pictures were presented for 8 seconds each with an intertrial interval (ITI) between 16.5 and 25.5 seconds (mean = 21 seconds). Startle probes were administered 2.5, 4.0, or 5.5 seconds after picture onset during picture presentation as well as 10 or 12 seconds after picture offset during the ITI. 75% of all trials contained startle probes during picture presentation (evenly distributed across each picture category), 12.5% of all trials contained startle probes during the ITI and 12.5% of the trials did not contain any startle probe. Two electrodes were placed under the left eye [65] to measure the electromyogram (EMG) activity of the orbicularis oculi muscle. The reference electrode was placed on the forehead, the ground electrode was placed on the processus mastoideus. BrainVision Analyzer 2 (Brain Products GmbH, Gilching, Germany) was used as analyzing software, to rectify, filter (Low Cutoff 28 Hz, High Cutoff 500 Hz, Notch 50 Hz), and smooth the signals offline (using a time constant of 50 ms). The difference between the highest peak 21 to 200 ms after and the average across 50 ms before startle probe presentation was taken as startle magnitude.

### Caffeine intervention

The above mentioned startle paradigm was embedded in a double-blind, placebo-controlled caffeine challenge study-design (as described in detail in Domschke et al., 2012 [67]). Briefly, caffeine intervention was performed by oral administration of 300 mg caffeine citrate (equivalent to 150 mg freebase caffeine; cf. [61], [139], [140]). Participants were given a placebo or caffeine capsule 60 minutes before starting the startle paradigm. Caffeine levels were determined by saliva test.

### Assessment of Childhood Trauma

All participants completed the German version of the Childhood Trauma Questionnaire [132], [133], comprising 28 items with a total sum score between a minimum of 25 and a maximum of 128 designed to retrospectively assess negative childhood experiences [133], [141], [142]. After instruction by the investigators, participants completed the CTQ on a computer in the laboratory and had the opportunity to ask questions concerning the questionnaire during the test session. The internal consistency of the German version of the CTQ sum score was found to be excellent (Cronbach́s α = 0.94; [143]). Also, the retest-reliability (r = 0.74–0.94) was found to be high [144]. The construct validity was comparable to the original English version [143]. A distinct convergence with other assessments of childhood maltreatment was observed [144]. For the German version of the CTQ, mean scores are only reported for different mental disorders and range between 40.1 in patients with anxiety disorders up to 63 in patients with borderline personality disorder [143].

### Statistical Analysis

To control for influences of potentially extreme scores, all analyzed startle data were checked for outliers (>2.5 SD; cf. [145]). To prevent statistical inference errors from non-normality of data and to reduce influences of potential outliers, CTQ score was logarithmized using the natural log with base *e* (cf. [146]).

To exclude a possibly confounding influence of *ADORA2A* 1976T>C and *5-HTTLPR* genotype distribution (see above), we evaluated frequencies of these genotypes in *COMT* Val158Met genotype groups by χ^2^ tests: *ADORA2A* 1976T>C and *5-HTTLPR* genotype distributions did not differ between *COMT* genotype groups (*ADORA2A*: χ^2^ = 6.75, *p* = 0.15; *5-HTTPLR*: χ^2^ = 1.14, *p* = 0.89), sex (*ADORA2A*: χ^2^ = 0.59, *p* = 0.74; *5-HTTPLR*: χ^2^ = 1.38, *p* = 0.50) or challenge conditions (*ADORA2A*: χ^2^ = 1.49, *p* = 0.48; *5-HTTPLR*: χ^2^ = 0.43, *p* = 0.81).

Possible differences regarding age and CTQ between sex (male vs. female participants), challenge condition (caffeine vs. placebo) and *COMT* Val158Met genotype groups were analyzed by means of T tests.

For genetic analyses, in a first step *COMT* Val158Met genotypes were coded as 0, 1 and 2 for the number of val alleles. Sex was coded as 0.5 for females and −0.5 for males, challenge condition as 0.5 for caffeine and −0.5 for placebo (cf. [147]). To exclude possible objectionable confounding effects, gene-environment correlations (rGE) were analyzed using bivariate correlation analysis.

Startle data were checked for zero responses and artifacts in each participant. Startle reactions with no detectable responses (fewer than 5 µV) were scored as zero. Artifacts were defined as spontaneous eye blinks during baseline or within 20 ms after startle probe onset and were scored as missing values. Participants with too many zero responses (more than 2.5 standard deviations above mean zero responses) or less than three acceptable startle responses in any picture category were excluded from data analysis. All startle responses were T-transformed within individual subjects in order to assure comparability of the data and to reduce interindividual variability (as described by [65];[56]). Despite the large variance in the untransformed raw data (startle (in µV) after presentation of unpleasant (M = 40.30, SD = 30.80), neutral (M = 37.18, SD = 28.27) and pleasant (M = 35.08, SD = 27.08) pictures) compared to the T-transformed data (see table 1), the basic ANOVA analysis was also conducted for raw data.

Influence of *COMT* Val158Met genotype on baseline startle response (ITI startle) was investigated using one-way ANOVA.

The affect-modulated startle responses were analyzed by ANOVA for repeated measures with picture category (unpleasant, neutral, and pleasant) as within-subjects factor and *COMT* genotype, sex and challenge condition (caffeine vs. placebo) as between-subjects factors. Pairwise comparisons of picture valence were assessed by means of post-hoc T-tests.

Influence of CTQ on ITI startle response and on response after presentation of pleasant, neutral and unpleasant pictures, respectively, was examined for the three *COMT* Val158Met genotypes separately by linear regression analyses.

For further genetic analyses based on the observed results yielded by the above mentioned statistics, *COMT* val/met and met/met genotypes were grouped together (0.5 = val/val vs −0.5 = val/met and −0.5 = met/met), reflecting a recessive model when assuming val to be the risk allele.

Also, the potentiations of the startle magnitude (T-transformed) after unpleasant IAPS picture presentation compared to neutral pictures (Diff_unpl-neutr_) and after neutral pictures compared to pleasant pictures (Diff_neutr-pl_) were calculated and used as dependent variables in the subsequent regression analyses. Based on the observed influences of sex, *COMT* Val158Met genotype and the interactive effects between *COMT* Val158Met genotype and CTQ (GxE), these effects on the potentiation of the startle magnitude were estimated by hierarchical multiple regression in a three-step procedure: The first included sex, grouped *COMT* Val158Met genotypes (see above) and logarithmized CTQ sum, in order to evaluate main effects on startle response. In a second step, a two-way interaction term for *COMT* Val158Met genotype and CTQ was added. Step 3 contained the two-way interaction of sex with *COMT* Val158Met genotype and CTQ sum and a three-way interaction with all three predictors (*COMT* Val158Met genotype, CTQ, sex). This order of variable entry enabled us to first investigate only main effects on startle magnitude potentiation. In a second step, we analyzed the increase in explained variance by inclusion of GxE. Thirdly, we tested for interactions with sex. Increase in explained variance (ΔR^2^) in successive regression steps was tested by means of F-test. For each regression step, Gaussian distribution of residual variances was confirmed by a post-hoc Kolmogorov-Smirnov test.

For all tests, a *p*-value <0.05 was considered statistically significant. All analyses were run with SPSS Version 18.0.
